# Retinoblastoma-Binding Protein 5 Regulates H3K4 Methylation Modification to Inhibit the Proliferation of Melanoma Cells by Inactivating the Wnt/*β*-Catenin and Epithelial-Mesenchymal Transition Pathways

**DOI:** 10.1155/2023/5093941

**Published:** 2023-02-21

**Authors:** Zhiqin Yang, Yue Jia, Shaojia Wang, Yongjun Zhang, Wen Fan, Xin Wang, Liang He, Xiaoyu Shen, Xiangqun Yang, Yi Zhang, Hongying Yang

**Affiliations:** ^1^Department of Gynecology, The Third Affiliated Hospital of Kunming Medical University (Tumor Hospital of Yunnan Province), Kunming 650118, China; ^2^Departments of Gynecology, The First Affiliated Hospital of Kunming Medical University, Kunming 650032, China; ^3^Departments of Reproduction, The Second Affiliated Hospital of Kunming Medical University, Kunming 650106, China

## Abstract

Histone 3 lysine 4 methylation (H3K4me), especially histone 3 lysine 4 trimethylation (H3K4me3), is one of the most extensively studied patterns of histone modification and plays crucial roles in many biological processes. However, as a part of H3K4 methyltransferase that participates in H3K4 methylation and transcriptional regulation, retinoblastoma-binding protein 5 (RBBP5) has not been well studied in melanoma. The present study sought to explore RBBP5-mediated H3K4 histone modification and the potential mechanisms in melanoma. RBBP5 expression in melanoma and nevi specimens was detected by immunohistochemistry. Western blotting was performed for three pairs of melanoma cancer tissues and nevi tissues. In vitro and in vivo assays were used to investigate the function of RBBP5. The molecular mechanism was determined using RT-qPCR, western blotting, ChIP assays, and Co-IP assays. Our study showed that RBBP5 was significantly downregulated in melanoma tissue and cells compared with nevi tissues and normal epithelia cells (*P* < 0.05). Reducing RBBP5 in human melanoma cells leads to H3K4me3 downregulation and promotes cell proliferation, migration, and invasion. On the one hand, we verified that WSB2 was an upstream gene of RBBP5-mediated H3K4 modification, which could directly bind to RBBP5 and negatively regulate its expression. On the other hand, we also confirmed that p16 (a cancer suppressor gene) was a downstream target of H3K4me3, the promoter of which can directly bind to H3K4me3. Mechanistically, our data revealed that RBBP5 inactivated the Wnt/*β*-catenin and epithelial-mesenchymal transition (EMT) pathways (*P* < 0.05), leading to melanoma suppression. Histone methylation is rising as an important factor affecting tumorigenicity and tumor progression. Our findings verified the significance of RBBP5-mediated H3K4 modification in melanoma and the potential regulatory mechanisms of melanoma proliferation and growth, suggesting that RBBP5 is a potential therapeutic target for the treatment of melanoma.

## 1. Introduction

Melanoma is the most lethal type [1] of skin tumor and originates from the malignant translation of melanocytes [2]. Melanocytes are derived from neuroectodermal cells and are widely distributed throughout the body. As a result, melanoma can occur ubiquitously, including skin, acra, mucosa, rectum, and uvea [3, 4]. In 2022, there are estimated 99,780 new skin melamoma patients and 7,650 deaths occurred in the United States [5]. Although targeted and immune-based therapeutic strategies have improved the survival rate of melanoma in recent years, 60–70% of melanoma patients still have no response to immune checkpoint inhibitors due to toxicity, drug resistance or other reasons [6]. In patients with metastatic melanoma, the 5-year survival rate is only approximately 23% [7]. Therefore, exploring the molecular mechanisms and discovering new strategies for melanoma are essential to improve the outcomes of melanoma.

Retinoblastoma-binding protein 5 (RBBP5), which is also known as SWD1 or RBQ3, is a protein coding gene and encodes a nuclear protein that belongs to a highly conserved subfamily of water displacement (WD)-repeat proteins. The encoded protein binds directly to retinoblastoma protein, which regulates cell proliferation. RBBP5, WDR5, ASH2L, and DPY30 are components of the WRAD complex, which is an H3K4 methyltransferase and participates in H3K4 methylation and transcriptional regulation [8–12]. The H3K4 methyltransferase family has similar structures, the SET domain for catalysis and the WRAD complex for regulation. The methyltransferase activity of the SET domain itself is weak, which has high methyltransferase activity only after binding with the WRAD complex [8–11]. Therefore, the deletion of RBBP5 may lead to a decrease in H3K4 methyltransferase activity and the degree of H3K4 methylation. In addition, previous studies have shown that RBBP5 is associated with prostate carcinoma [13], hepatocellular carcinoma [14], multiple myeloma [15], gliomas [16], etc.

Histone modifications can change the structure and affect gene transcription. H3K4me, especially H3K4me3, is a well-known epigenetic mark of histone modification. In recent years, H3K4me3 has been found to play a crucial role in many cancers, including breast carcinoma [17], cervical cancer [18], renal carcinoma [19, 20], colon cancer [21], ovarian cancer [22], and hepatocellular carcinoma [23]. H3K4me3 is associated not only with transcriptional activation but also with suppressed gene expression [24, 25]. However, the roles of H3K4me3 and RBBP5 in melanoma remain unknown.

In the present study, we found that RBBP5 was a tumor suppressor factor in human melanoma,which was downregulated in melanoma tissues and cells. Functional studies showed that overexpression of RBBP5 could enhance the expression of H3K4me3 and inhibit the proliferation, migration and invasion of melanoma cells. We explored the signalling pathway related to RBBP5 and found that RBBP5 could affect the progression of melanoma through multiple signalling pathways, including Wnt/*β*-catenin and epithelial-mesenchymal transition (EMT). In addition, H3K4me3 could directly bind with the promoter of the tumor suppressor gene p16 and increase its expression. Our study suggested that RBBP5 plays an important role in tumor progression in melanoma and might be a potential therapeutic target for the treatment of melanoma.

## 2. Materials and Methods

### 2.1. Cell Culture

A375 (melanoma cell lines, Procell, CL-0014) was purchased from Procell Life Science & Technology Co. Ltd. (Hyderabad, India). A2058 (melanoma cell lines) and HaCat (normal epidermal cell line) cells were gifts from the biological treatment centre of the Third Affiliated Hospital of Kunming Medical University. All cells were cultured with Dulbecco's Modified Eagle Medium (DMEM, Gibco, c11995500BT; Waltham, MA, USA) supplemented with 10% fetal bovine serum (FBS, BI, 04-001-1ACS) and 1% penicillin/streptomycin (NCM, C125C5) at 37°C in an incubator with 5% CO_2_.

### 2.2. Tissue Specimens

Three pairs of nevi, melanoma, and adjacent tissues were obtained from patients at the Third Affiliated Hospital of Kunming Medical University and stored at −80°C refrigerator. In addition, 106 cases of paraffin-embedded melanoma tissues, 100 cases of paraffin-embedded adjacent specimens, and 23 cases of paraffin-embedded nevi tissues were also obtained from the Third Affiliated Hospital of Kunming Medical University. None of the patients were treated with chemotherapy or radiotherapy before surgery. This research was approved by the Ethics Committee of Kunming Medical University.

### 2.3. Western Blotting

After washing three times with phosphate-buffered saline (PBS, Corning, 21-040-CVC, NY, USA), cell and tissue samples were lysed on ice with cell lysis buffer (Beyotime, P0013, Shanghai, China), which was preadded with 50× protease and phosphatase inhibitor (Beyotime, P1046, Shanghai, China). After 30 min, the samples were centrifuged at 12000 rpm for 10 min at 4°C. The protein concentration was determined with a bicinchoninic acid (BCA) protein assay kit (Beyotime, P0012, Shanghai, China). Isolated proteins were separated by 8%–12% sodium dodecyl sulfate-polyacrylamide gel electrophoresis (SDS-PAGE) and transferred to polyvinylidene difluoride (PVDF) membranes (Bioleader, BSP0161; Seoul, Korea) by electrophoresis at 220 mA for 110 min. Then, the membranes were blocked with 1% (W/V) bovine serum albumin (BSA) or nonfat milk for 2 h at room temperature and incubated overnight at 4°C with the following primary antibodies: WSB2 (Proteintech, 12124-2-AP, 1 : 100; Rosemont, IL, USA), RBBP5 (Thermo Fisher Scientific, PA5-63522, 1 : 1000; Shanghai, China), H3K4me3 (Abcam, ab8580, 1 : 100; Cambridge, UK), P16 (Abcam, ab51243, 1 : 5000), *β*-catenin (Abcam, ab32572, 1 : 5000), c-myc (CST, 13987, 1 : 1000; Louisville, KY, USA), E-cadherin (CST, 3195, 1 : 1000), N-cadherin (CST, 13116, 1 : 1000), MMP-7 (Abcam, ab216631, 1 : 300), and glyceraldehyde-3-phosphate dehydrogenase (GAPDH) (Abcam, ab181602, 1 : 5000). The membrane was then incubated with horseradish peroxidase (HRP)-conjugated secondary antibodies (SAB, L3012, L3032) at room temperature for 1 h. Membranes were exposed after incubation with electrochemiluminescence (ECL) substrate (Biosharp, BL520B; Hangzhou, China) for 1 min. Image J software was used for semiquantitative analysis.

### 2.4. Quantitative Real-Time Polymerase Chain Reaction (qRT-PCR)

Total RNA was separated by TRIzol reagent (Invitrogen, 15596026; Shanghai, China). Cell samples were treated with chloroform for 5 min and centrifuged for 15 min at 4°C and 12000 rpm. Then, the upper aqueous phase was collected, and an equal volume of isopropanol was added. The samples were allowed to stand at −20°C for 30 min and centrifuged for 15 min at 12000 rpm at 4°C. Then, the RNA was washed with 75% ethanol, dried at room temperature, and dissolved in RNase-free water. RNA reverse transcription was performed to synthesize the first strand of cDNA using a Fast RT Kit (with gDNA) (Tiangen, KR116-02; Beijing, China) according to the manufacturer's instructions. Then, qRT-PCR was performed using SuperReal PreMix Plus (SYBR Green) (Tiangen, FP205-02) and a 7500 real-time PCR system (ABI-7500, Applied Biosystems, Waltham, MA, USA). The reaction conditions were as follows: 15 min at 95°C, followed by 40 cycles of 10 s at 95°C and 32 s at 60°C. GAPDH was used as an internal control. The relative expression of the mRNA was calculated by the 2^−ΔΔ*Ct*^ method. The primer sequences were as follows: GAPDH: F5′-TCTCTGCTCCTCCTGTTCGA-3′ and R5′-GCGCCCAATACGACCAAATC-3′; RBBP5: F5′-TTCTTTATGCTGGAGCCGA-3′ and R5′-GAAAGAACATCCCACTGTGAC-3′.

### 2.5. Cell Counting Kit-8 (CCK8) Assay

A CCK8 assay was performed to measure cell proliferation ability. A total of 2000 cells were seeded into a 96-well plate and incubated at 37°C and 5% CO_2_. Then, 100 *µ*l of serum-free medium containing 10 *µ*l CCK8 reagent (APE × Bio, K1018) was added to each well every day. After incubation at 37°C and 5% CO_2_ for 2 h, the absorbance was measured at 450 nm.

### 2.6. Wound Healing Assay

Cells were seeded into 6-well culture plates at a density of 1 × 10^6^ cells, until they reached 90%. Linear wounds were created using 100 *µ*l of tips, and cells were cultured for 24 h and 48 h in serum-free medium at 37°C and 5% CO_2_. Images were taken with a microscope, and the wound healing ability was examined by calculating the wound area.

### 2.7. Migration and Invasion Assays

The invasion/migration ability of the cells was measured in a Boyden chamber (Corning, 3422) with or without Matrigel (Corning, 356234). A total of 1 × 10^5^ A375 cells and 3 × 10^4^ A2058 cells were suspended in 200 *µ*l of serum-free medium and added to the upper chamber, and 600 *µ*l of complete medium containing 20% fetal bovine serum (FBS) was added to the lower chamber. After incubation for 24 h at 37°C and 5% CO_2_ in an incubator, the cells on the top surface of the membrane were removed, and the cells that had migrated or invaded to the lower surface of the membrane were fixed with methyl alcohol for 15 min and stained with 0.2% crystal violet for 30 min. The cells were photographed with a microscope, and the number of cells was counted by Image J.

### 2.8. Colony Formation Assay

For the colony formation assay, 500 cells were seeded in a 6-well plate, and the medium was changed every 3 days. The study was terminated when colony formation was visible to the naked eye. After washing the cells three times with PBS, the cells were fixed with methyl alcohol for 15 min and stained with 0.2% crystal violet for 30 min. Visible colonies were photographed and counted with Image J.

### 2.9. Plasmid Construction, Lentiviral Infection, and Transfection

The lentiviral vector containing small hairpin RNAs (shRNAs) and RBBP5 overexpression plasmids were purchased from Syngen Tech Co. Ltd. (Beijing, China). A375 and A2058 cells were transfected according to the manufacturer's instructions, and then stable lines that expressed RBBP5 and shRBBP5 (shRBBP5-1 and shRBBP5-2) were screened using puromycin (0.5 *µ*g/*µ*l) for 10 days. The target sequences were as follows: shRBBP5-1 (5′-TGGAGCCGAGATGGTCATAAA-3′) and shRBBP5-2 (5′-TCATTGTACCCAGCGTCATTT-3′). The shWSB2 plasmids were purchased from Genechem Co. Ltd. (Shanghai, China). The target sequences were as follows: shWSB2-1 forward: 5′-CTTCGAAGTTTCCTAACAA-3′ and reverse: 5′-GAAGCTTGCCCGGATTGTT-3′;shWSB2-2 sense forward: 5′-CGGCTTCTTACGATACCAA-3′ and reverse: 5′-GCCGAAGAATGCTATGGTT-3′. The shWSB2 plasmids were verified by western blotting.

### 2.10. Chromatin Immunoprecipitation (ChIP) Assay

ChIP was performed using a Simple ChIP® Enzymatic Chromatin IP Kit (magnetic beads) (CST, 9003) according to the manufacturer's instructions. In brief, cells were seeded into a 15 cm culture dish until the density reached 90%. To cross-link the protein with DNA, 540 *µ*l 37% formaldehyde was added to each 15 cm dish, which contained 20 ml of medium (making the final concentration of formaldehyde 1%), and the dish was rotated and incubated at room temperature for 10 minutes. Then, 2 ml of 10× glycine was added, and the dish was slightly vortexed and incubated at room temperature for 5 minutes, and it was washed with 1x PBS two times. Then, 2 mL of frozen 1× PBS + 200× PIC was added, and then the dish was centrifuged at 4°C and 2000× g for 5 minutes. Next, nuclear preparation and chromatin digestion were performed, and fragmented chromatin was obtained by nuclease and sonication. Chromatin immunoprecipitation was performed, and sufficient 1× chip buffer was prepared to dilute the digested chromatin. Then, 10 *µ*l rabbit anti-histone H3 (CST, 4620) was added as a technical positive control, 2 *µ*l normal rabbit immunoglobulin G (IgG) (CST, 2729) was added as a negative control, and 2 *µ*l rabbit antiH3K4me3 antibody (Abcam, ab8580) was added. The immunoprecipitation (IP) samples were rotated and incubated at 4°C overnight. Then, 30 *μ*l of Protein G Magnetic Beads (CST, 9006) was added to each IP reaction, rotated, and incubated for 2 hours at 4°C. After reverse cross-linking and DNA purification, immunoprecipitated DNA was quantified by real-time PCR using SimpleChIP® Universal qPCR Master Mix (CST, 88989) with primers for p16 binding sites in the p16 promoter (forward primer: 5′-AGCACTCGCTCACGGCGTC-3′ and reverse primer: 5′-CTGTCCCTCAAATCCTCTGGA-3′) and RPL30 (CST, 7014). Fold enrichment was calculated based on the threshold cycle (CT) value of the IgG control using the comparative CT method. Input percentage = 2% × 2 (C[T] 2% input sample − C[T] IP sample) C[T] = CT = threshold period of PCR.

### 2.11. Co-immunoprecipitation (Co-IP)

After washing three times with PBS, cells and tissue samples were lysed on ice with cell lysis buffer, which was preadded with protease and phosphatase inhibitors. After 30 min, the samples were centrifuged at 4°C and 12000 rpm for 10 min. A total of 100 *µ*l of protein lysate was used as an input control (positive control). Then, 2 *µ*l WSB2 antibody was added to 200 *µ*l protein lysate, 2 *µ*l normal rabbit IgG antibody was added to another 200 *µ*l protein lysate as a negative control, and the sample was rotated and incubated for 24 hours at 4°C. Then, 400 *µ*l phosphate-buffered saline with Tween PBST (1× PBS + 0.5% Tween-20, pH 7.4) was used to wash the Protein A/G Magnetic Beads (MCE, HY-K0202), and the beads were separated by a magnetic rack. The above step was repeated two times. The antigen antibody mixture was added to the pretreated beads and incubated at 4°C for 3 hours to obtain the antigen-antibody-bead mixture. The antigen-antibody-bead mixture was adsorbed with a magnetic rack, and then 5x sodium dodecyl sulfate (SDS) loading buffer (including the input control) was added and boiled at 95°C for 10 min. Western blotting was used to verify the interaction between proteins.

### 2.12. Xenograft Mouse Model

The animal studies were approved by the Animal Ethics Committee of Kunming Medical University. Six- to eight-week-old female BALB/c nude mice were purchased from SPF Biotechnology Co. Ltd. (Beijing, China). For the preliminary experiment, ten mice were randomly divided into two groups (*n* = 5 per group), and 2 × 10^6^ RBBP5 overexpression or vector A375 cells were injected subcutaneously into each mouse. In a follow-up experiment, thirty mice were randomly divided into four groups. The first two groups had ten mice in each group, and 2 × 10^6^ RBBP5 knockdown or vector A375 cells were injected subcutaneously. The latter two groups had 5 mice in each group, and 2 × 10^6^ RBBP5 overexpression or vector A375 cells were injected subcutaneously. The volume of the tumor was measured by the equation (*L* × *W*^2^)/2. At the end of the experiment, the tumors were quantified.

### 2.13. Immunohistochemistry (IHC)

The paraffin-embedded tissue or formalin-fixed xenograft tumor samples were cut into 4 *µ*m-thick sections. Sections were deparaffinized with xylene, rehydrated with ethanol, and then treated with 3% H_2_O_2_ for 25 min at room temperature to inhibit the activity of endogenous peroxidase. Sections were blocked with 3% BSA at room temperature for 30 min to block nonspecific binding and then incubated with primary antibodies overnight, and HRP-labelled goat antirabbit IgG was added (Servicebio, GB23303; Woburn, MA, USA). The primary antibodies were as follows: WSB2 (Peproteintech, 12124-2-AP; Rosemont, IL, USA), RBBP5 (Thermo Fisher Scientific, PA5-63522), H3K4me3 (Abcam, ab8580), P16 (Abcam, ab51243), and N-cadherin (CST, 13116). DAB (3,3′-diaminobenaidine) and haematoxylin were used for chromogen and counterstaining. Immunostaining results of RBBP5, WSB2, N-cadherin and p16 were evaluated independently by two pathologist. In each case, four representative areas were selected and observed at 400x magnification. For each case, the average of the single counts of positive tumor cells per field was considered. The final immunohistochemical score was based on the proportion of positive tumor cells and intensity of staining. The staining intensity was as follows: 0 = no staining, 1 = weak staining = light yellow, 2 = moderate staining = yellow brown, and 3 = strong staining = brown. The percentage of positive staining was as follows: 0 = 0%–5%, 1 = 15%–20%, 2 = 25%–50%, 3 = 50%–75%, and 4 = 75%–100%. A score <4 was defined as low expression, and a score ≥4 was defined as high expression.

### 2.14. Flow Cytometry

For the cell cycle assay, 1 × 10^6^ cells were seeded into a 6-well plate for 24 h and then harvested and washed twice with PBS. A total of 1 ml of 75% cold ethanol was used to fix the cells, and a Cell Cycle and Apoptosis Analysis Kit (Beyotime, C1052) was used to stain the cells for 30 min in a 37°C dark and warm bath. The cells were run on a FACS Calibur flow cytometer (BD Biosciences, Haryana, India). Data were analyzed with FlowJo software.

### 2.15. Statistical Analysis

Statistical analysis of all data was performed with SPSS 20.0 and GraphPad Prism 8.0 software. Data are presented as mean ± standard deviation (SD). All experiments were performed at least three times. Student's *t-*test was used to compare differences between two groups. One-way analysis of variance (ANOVA) was used to compare differences among multiple groups. *p* < 0.05 was considered statistically significant. Significance is presented as follows: ^*∗*^*p* < 0.05,  ^*∗∗*^*p* < 0.01, and ^*∗∗∗*^*p* < 0.001.

## 3. Results

### 3.1. WSB2 Was Bound Directly to RBBP5 and Negatively Regulated It

**Figure 1:**
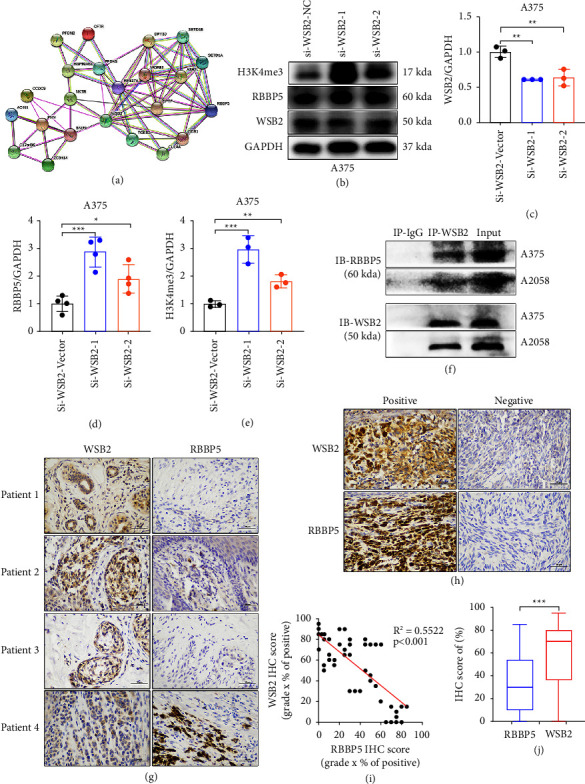
WSB2 was bound directly to RBBP5 and negatively regulated it. (a) STRING (“https://string-db.org”), an online database, showed that WSB2 was bound to the RBBP5 protein. (b, c) Western blotting was used to verify WSB2 protein levels after WSB2 knockdown. (b, d, e) Western blotting was used to explore the changes in RBBP5 and H3K4me3 protein after WSB2 knockdown. (f) Co-IP and western blotting were performed to evaluate the interaction between WSB2 and RBBP5. Data are represented as mean ± SD of three independent experiments. ^*∗*^*p* < 0.05,  ^*∗∗*^*p* < 0.01, and ^*∗∗∗*^*p* < 0.001; ns, no significance. (g, h) Immunohistochemical staining of RBBP5 and WSB2 in 104 cases of melanoma tumor tissues. Representative images are shown. Scale bar: 50 *µ*m. The correlation between RBBP5 and WSB2 was calculated (i, j). *n* = 104, Pearson chi-square test.

The research results of our group showed that WSB2 was significantly increased in melanoma and promoted the proliferation and migration of melanoma cells [26]. Search Tool for the Retrieval of Interacting Genes/Proteins (STRING) (https://cn.string-db.org/), an online database, was used to predict the downstream targets of WSB2, and the results showed that WSB2 was bound to the RBBBP5 protein (Figure 1(a)). Co-IP was used to confirm the direct protein association between WSB2 and RBBP5 (Figure 1(f)). In addition, we found that WSB2 knockdown efficiently enhanced RBBP5 and H3K3me3 expression in melanoma cells (Figures 1(b)–1(e)). To further explore the correlation between WSB2 and RBBP5, we performed immunohistochemical staining on 104 melanoma paraffin sections (Figure 1(g)). The results were classified as negative or positive (Figures 1(h) and 1(j)). Positive WSB2 staining was observed mainly in the cytoplasm and partly in the nucleus. Positive RBBP5 was observed mainly in the cytoplasm and nucleus. The IHC score showed that RBBP5 expression was negatively correlated with WSB2 expression (Figure 1(i)). These experiments verified that WSB2 could bind with RBBP5 and then negatively regulate RBBP5 and H3K4me3 protein expression.

### 3.2. RBBP5 Was Downregulated in Melanoma

**Figure 2:**
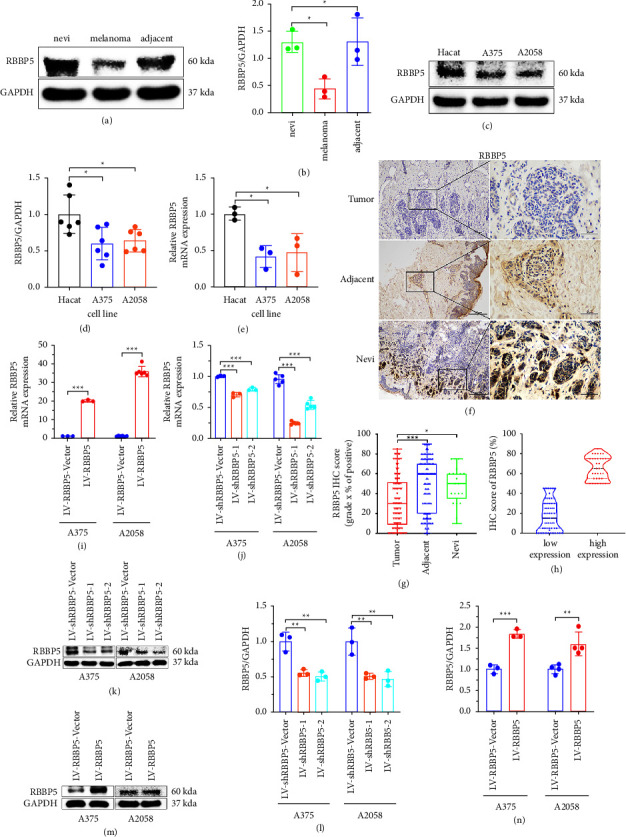
RBBP5 was downregulated in melanoma. (a, b) Western blot to explore the expression of RBBP5 in three pairs of melanoma tissue compared with nevi and adjacent tissue. (c, d) Western blotting and (e) qRT-PCR were performed to verify the mRNA and protein expression of RBBP5 in two melanoma cell lines (A375 and A2058) compared with a human immortalized keratinocyte (HaCaT) cell line. (f) IHC of RBBP5 was performed in 106 cases of melanoma tumor tissues, 100 cases of adjacent tissues, and 23 cases of nevi tissues. Representative images with different tissues stained are shown. Scale bar: 200 *µ*m and 50 *µ*m. (g, h) The relative score of RBBP5 was quantified by the grade of staining intensity and the percentage of positively stained cells. A375 and A2058 cells were transfected with empty vector (shRBBP5-vector) or shRNAs targeting RBBP5 (shRBBP5), and the relative mRNA level of RBBP5 was measured by qRT-PCR. GAPDH was used as an internal control (j). The protein expression of RBBP5 was detected by western blotting. GAPDH was used as a loading control (k, l). A375 and A2058 cells were transfected with RBBP5 empty vector (RBBP5-vector) or RBBP5 overexpression lentivirus (RBBP5), and transfection efficiency was verified by q-PCR (i) and western blotting (m, n). GAPDH was used as a loading control. Data are represented as mean ± SD of three independent experiments. ^*∗*^*p* < 0.05,  ^*∗∗*^*p* < 0.01, and ^*∗∗∗*^*p* < 0.001; ns, no significance.

To explore the potential role of RBBP5 in melanoma, the expression of RBBP5 in melanoma was investigated by WB, IHC and qRT-PCR. First, WB results showed that the expression of RBBP5 was significantly decreased in melanoma tissue compared with nevi and adjacent tissue (*p* < 0.05, Figures 2(a) and 2(b)). Second, IHC was performed in 106 cases of melanoma tumor tissues, 100 cases of adjacent tissues, and 23 cases of nevi tissues to identify the potential difference among different groups. Positive RBBP5 staining was detected in the nucleus and cytoplasm. The percentage of positive RBBP5 staining was 12.3% (13/106) in tumor tissues, 24% (24/100) in adjacent tissues, and 21.8% (5/23) in nevi tissues. Moreover, the average RBBP5 IHC score of tumor tissues was lower than the average RBBP5 IHC score of adjacent tissues and nevi tissues (Figures 2(f)–2(h)). Third, the qRT-PCR and WB results also showed that the expression of RBBP5 was significantly downregulated in two melanoma cell lines (A375 and A2058) compared with a human immortalized keratinocyte (HaCaT) cell line (Figures 2(c)–2(e)). Together, our study showed that the expression of RBBP5 was significantly downregulated in melanoma tissue and cells. To explore the function of RBBP5 in melanoma, we constructed stable cell lines with RBBP5 knockdown and overexpression in A375 and A2058 cells (Figures 2(i)–2(n)).

### 3.3. RBBP5 Positively Regulated the Expression of H3K4me3 and P16

**Figure 3:**
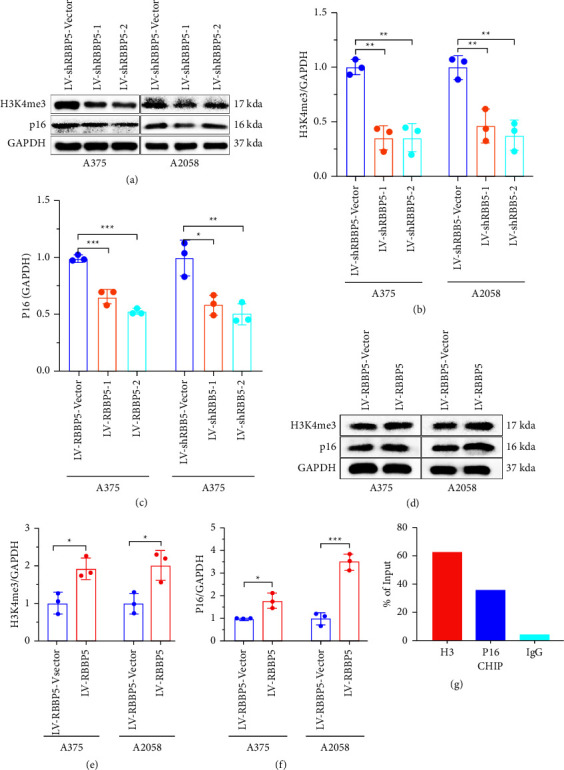
RBBP5 positively regulated the expression of H3K4me3 and P16, and H3K4me3 could bind to the P16 promoter. (a–c) RBBP5 knockdown and (d–f) RBBP5 overexpression in A375 and A2058 cells, and the expression levels of H3K4me3 and p16 were detected by western blotting. GAPDH was used as an internal control. (g) ChIP was used to verify the interaction of H3K4me3 and p16. Data are represented as mean ± SD of three independent experiments. ^*∗*^*p* < 0.05,  ^*∗∗*^*p* < 0.01, and ^*∗∗∗*^*p* < 0.001; ns, no significance.

According to our previous study, we found that the expression of RBBP5 and H3K4me3 was notably downregulated after WSB2 knockdown. To explore the function of RBBP5 in melanoma, we constructed stable cell lines with RBBP5 knockdown and overexpression in A375 and A2058 cells (Figures 2(i)–2(n)). Our results indicated that the expression of H3K4me3 and p16 was significantly inhibited after RBBP5 knockdown (Figures 3(a)–3(c)). In contrast, the expression of H3K4me3 and p16 was significantly increased after RBBP5 was overexpressed (Figures 3(d)–3(f)). Because H3K4me3 can bind directly to the promoter of genes and mediate transcriptional activation, we further investigated the interaction of H3K4me3 and p16 by chromatin immunoprecipitation (ChIP). We observed that H3K4me3 can bind to the promoter of p16 and then upregulate p16 expression (Figure 3(g)). Together, our study showed that RBBP5 could upregulate the level of H3K4me3, which could bind to the promoter of p16 and activate transcription of p16.

### 3.4. RBBP5 Inhibits the Proliferation, Migration, and Invasion of Melanoma In Vitro

**Figure 4:**
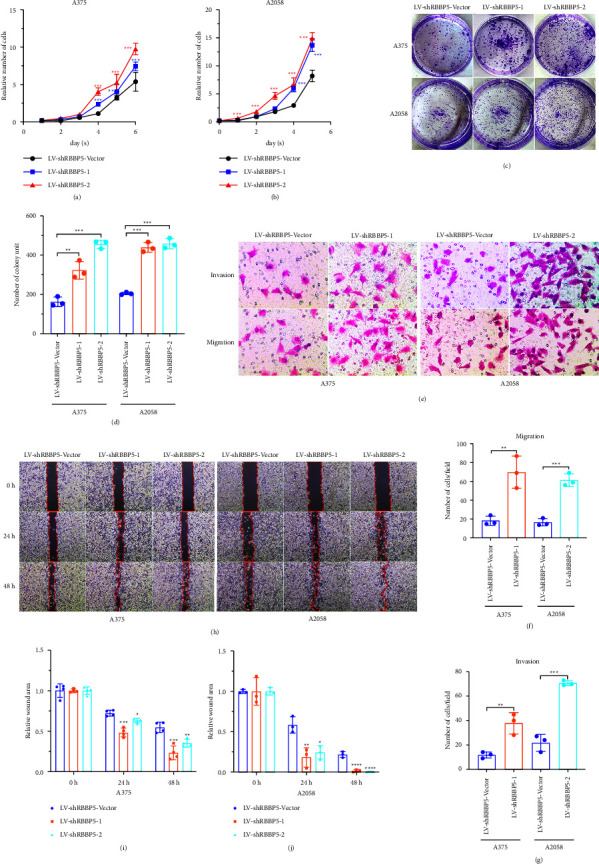
RBBP5 knockdown promoted the progression of melanoma in vitro. CCK8 assay was performed to measure the proliferation ability of RBBP5 knockdown in A375 (a) and A2058 (b) cells. Representative pictures (c) and quantification (d) of colony formation ability was shown in A375 and A2058 cells after RBBP5 knockdown. Representative pictures (e, f) and quantification (f-g) of the migration and invasion ability was shown in A375 and A2058 cells after RBBP5 knockdown. Data are represented as the mean ± SD of three independent experiments. ^∗^*p* < 0.05, ^∗∗^*p* < 0.01, ^∗∗∗^*p* < 0.001.

**Figure 5:**
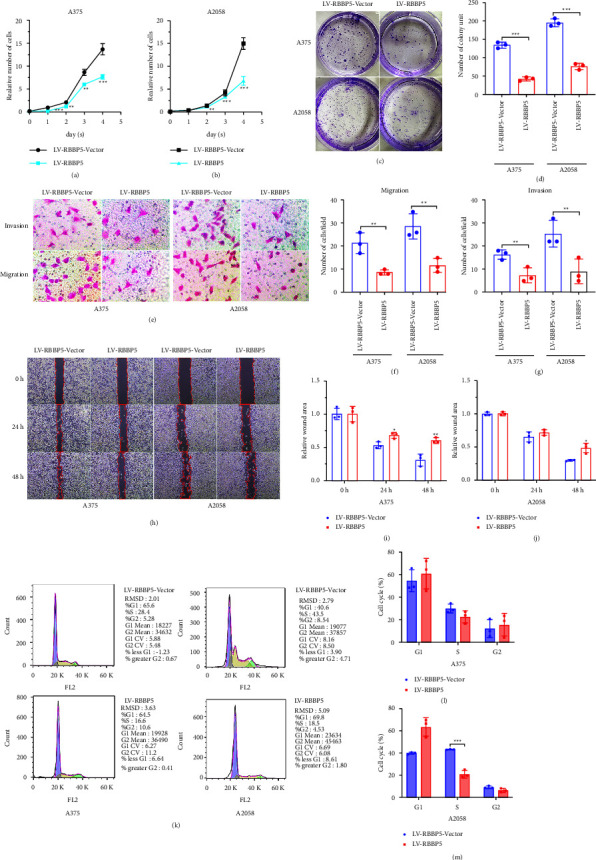
RBBP5 inhibited the progression of melanoma and arrested the cell cycle in in vitro. CCK8 assay was performed to measure the proliferation ability of RBBP5-overexpressing in A375 (a) and A2058 (b) cells. Representative pictures (c) and quantification (d) of colony formation ability was shown in A375 and A2058 cells after RBBP5-overexpressed. Representative pictures (e, h) and quantification (f, g, i, j) of the migration and invasion ability was shown in A375 and A2058 cells after RBBP5-overexpressed. FACS analysis of propidium iodide-stained cells was used to obtain cell cycle profiles, and flow cytometry was used to analyse the cell cycle in RBBP5-overexpressing A375 (k left, l) and A2058 (k right, m) cells. Data are represented as the mean ± SD of three independent experiments. ^*∗*^*p* < 0.05,  ^*∗∗*^*p* < 0.01, and ^*∗∗∗*^*p* < 0.001.

To further verify whether RBBP5 could inhibit the progression of melanoma cells in vitro, CCK8, wound healing, colony formation, migration, and invasion experiments were performed. Our results showed that the proliferation (Figures 4(a) and 4(b)), colony formation (Figures 4(c) and 4(d)), migration (Figures 4(e) and 4(f)), invasion (Figures 4(e) and 4(g)), and wound healing (Figures 4(h)–4(j)) abilities of A375 and A2058 cell lines were significantly enhanced after RBBP5 knockdown. In contrast, proliferation (Figures 5(a) and 5(b)), colony formation (Figures 5(c) and 5(d)), migration (Figures 5(e) and 5(f)), invasion (Figures 5(e) and 5(g)), and wound healing (Figures 5(h)–5(j)) in A375 and A2058 cell lines were markedly inhibited after RBBP5 was overexpressed. To explore the effect of RBBP5 on the cell cycle, we performed flow cytometry to analyze the cell cycle distribution. The results showed that overexpression of RBBP5 caused the percentage of cells in the S phase to decrease and the percentage of cells in the G0/G1 phase to increase in A375 melanoma cells (Figures 5(k) and 5(l)) compared with control cells. Similar results were observed in A2058 cells (Figures 5(k) and 5(m)). Taken together, our study indicated that RBBP5 could inhibit the progression of melanoma cells in vitro and inhibit the G1/S transition.

### 3.5. RBBP5 Downregulated the Activity of the Wnt/*β*-Catenin and EMT Signalling Pathways

**Figure 6:**
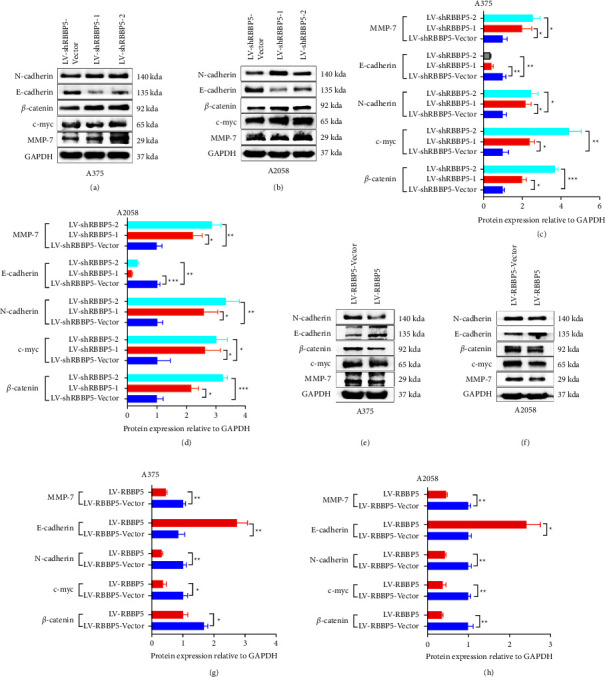
RBBP5 inhibits the Wnt/*β*-catenin signalling pathway and EMT. (a–d) The effect of RBBP5 silencing on the Wnt/*β*-catenin signalling pathway and EMT marker expression was evaluated by western blotting in A375 and A2058 cells. (e–h) The effect of RBBP5 overexpression on the Wnt/*β*-catenin signalling pathway and EMT marker expression was evaluated by western blotting in A375 and A2058 cells. Data are represented as mean ± SD of three independent experiments. ^*∗*^ *p* < 0.05,  ^*∗∗*^ *p* < 0.01, and ^*∗∗∗*^*p* < 0.001; ns, no significance.

A previous study showed that WSB2 could activate the Wnt/*β*-catenin signalling pathway and promote the proliferation of melanoma cells. To explore the molecular mechanism of RBBP5 in melanoma cells, we analyzed the expression of *β*-catenin and c-myc, two marked target genes of the Wnt/*β*-catenin signalling pathway, in A375 and A2058 cells. We observed that upon RBBP5 knockdown in A375 and A2058 cells, the expression of *β*-catenin and c-myc was upregulated, as shown by western blotting (Figures 6(a)–6(d)). We also observed that two well-known proteins of EMT, N-cadherin and MMP-7, were upregulated, and E-cadherin was downregulated in RBBP5 knockdown A375 and A2058 cells (Figures 6(a)–6(d)). The results were contrary to RBBP5-overexpressing cell lines A375 and A2058 (Figures 6(e)–6(h)). Together, our study demonstrated that RBBP5 could inactivate the Wnt/*β*-catenin signalling pathway and inhibit EMT.

### 3.6. RBBP5 Inhibits the Proliferation of Melanoma In Vivo

**Figure 7:**
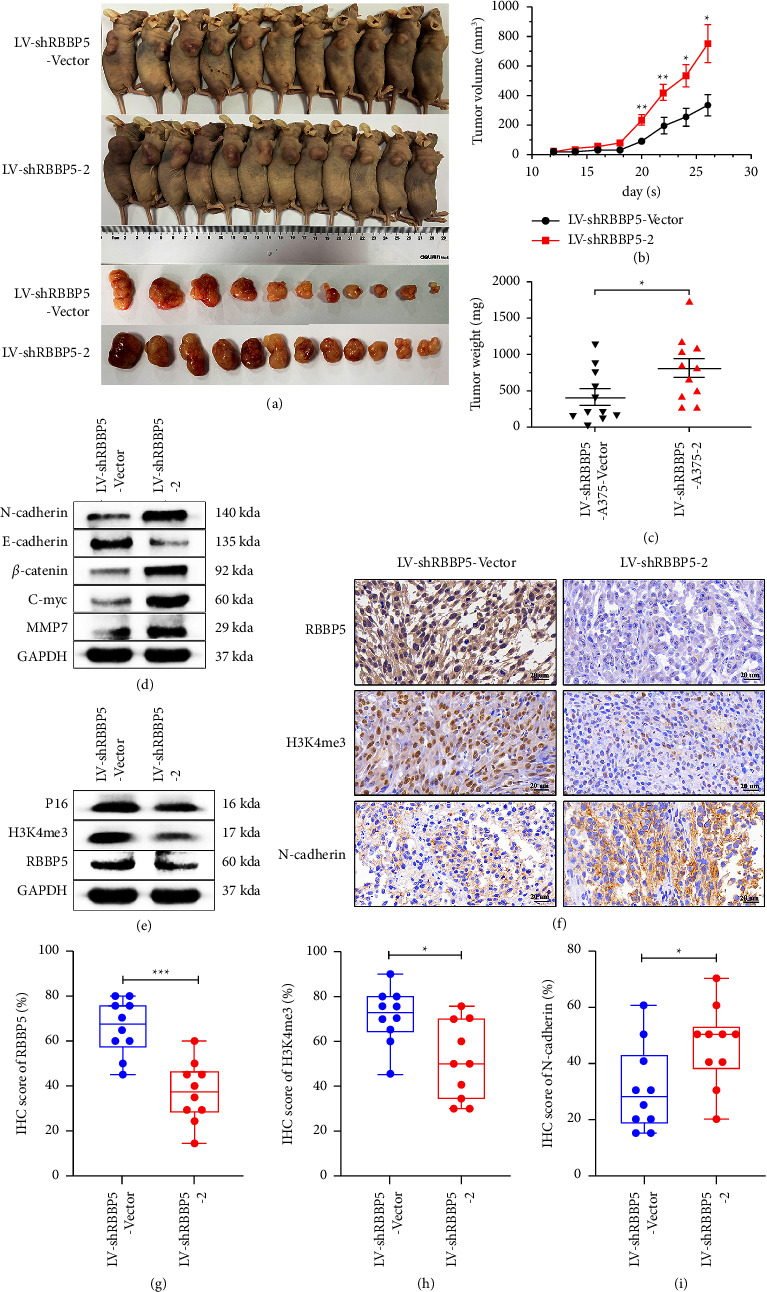
RBBP5 knockdown promotes melanoma growth in vivo. (a) Images of nude mice at the end of experiment; (b) Tumor volume (mm3) was measured to draw tumor growth curves and (c) tumour weight (mg) were measured to evaluate the growth of xenograft tumors; (d) Western blotting was used to detect the expression of marker proteins in the Wnt/*β*-catenin signalling pathway (*β*-catenin and c-myc) and EMT (N-cadherin, E-cadherin, and MMP-7). (e) Western blotting was used to detect the expression of H3K4me3 and p16 in mouse xenograft tumour tissues; IHC was used to detect the expression of RBBP5, H3K4me3, and N-cadherin in mouse xenograft tumour tissues. (f) Representative images; (g–i) quantification results were shown. Scale bar: 20 *μ*m. Data are represented as the mean ± SD. ^*∗*^*p* < 0.05,  ^*∗∗*^ *p* < 0.01, and ^*∗∗∗*^*p* < 0.001.

**Figure 8:**
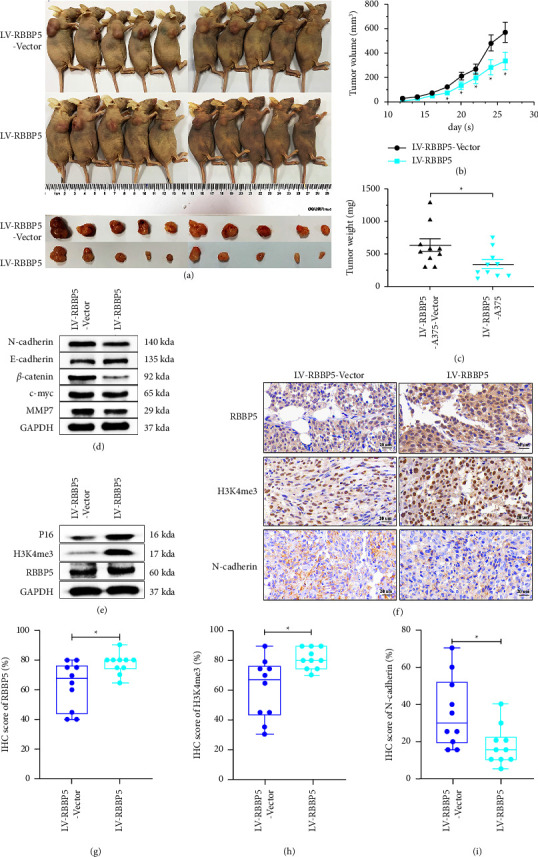
RBBP5 suppresses the growth of melanoma in vivo. (a) Images of nude mice at the end of experiment; (b) tumor volume (mm3) was measured to draw tumor growth curves and (c) tumour weight (mg) was measured to evaluate the growth of xenograft tumors; (d) Western blotting was used to detect the expression of marker proteins in the Wnt/*β*-catenin signalling pathway (*β*-catenin and c-myc) and EMT (N-cadherin, E-cadherin, and MMP-7). (e) Western blotting was used to detect the expression of H3K4me3 and p16 in mouse xenograft tumour tissues; IHC was used to detect the expression of RBBP5, H3K4me3, and N-cadherin in mouse xenograft tumour tissues. (f) Representative images and (g–i) quantification results were shown. Scale bar: 20 *μ*m. Data are represented as the mean ± SD. ^*∗*^*p* < 0.05,  ^*∗∗*^*p* < 0.01, and ^*∗∗∗*^*p* < 0.001.

To investigate the impact of RBBP5 on the tumorigenic capacity of melanoma cells in vivo, we subcutaneously injected RBBP5-overexpressing and RBBP5-silenced A375 cells into female nude mice. We observed that RBBP5 knockdown increased the subcutaneous xenograft volume and tumor weight compared with the control group (Figures 7(a)–7(c)). There was no significant difference in body weight of mice (Supplementary Figure S3). In contrast, overexpression of RBBP5 in A375 cells resulted in a marked decrease in the tumor-initiating ability and tumor weight (Figures 8(a)–8(c)). In addition, the western blot results showed that the expression of the RBBP5 downstream proteins H3K4me3 and p16 was decreased in the RBBP5 knockdown group (Figure 7(e), Supplementary Figure S1). Furthermore, the expression of*β*-catenin and c-myc in the Wnt/*β*-catenin signalling pathway was increased in the RBBP5 knockdown group in xenogeneic tumor tissues of nude mice, and N-cadherin and MMP-7 in EMT also increased (Figure 7(d), Supplementary Figure S1). We obtained the opposite results in the RBBP5-overexpressing group of xenogeneic tumor tissues (Figures 8(d) and 8(e), Supplementary Figure S2). These results were in agreement with our previous cell experiments. Immunohistochemical staining of xenogeneic tumor tissues showed that RBBP5 knockdown significantly increased the expression of N-cadherin and decreased the expression of H3K4me3 (Figures 7(f)–7(i)), which was opposite to the RBBP5 overexpression group (Figures 8(f)–8(i)). Together, our research suggested that RBBP5 could suppress the tumor growth and EMT of melanoma cells in vivo.

## 4. Discussion

RBBP5 is a protein coding gene. In embryonic stem (ES) cells, RBBP5 plays a crucial role in differentiation potential, particularly along the neural lineage, regulating gene induction and H3 “Lys-4” methylation at key developmental loci, including those mediated by retinoic acid (by similarity). Components or associated components of some histone methyltransferase complexes regulate transcription through recruitment of those complexes to gene promoters [27]. As part of the MLL1/MLL complex, RBBP5 is involved in mono-, di-, and trimethylation at “Lys-4” of histone H3 [28]. Histone H3 “Lys-4” methylation represents a specific tag for epigenetic transcriptional activation [28]. In association with ASH2L and WDR5, RBBP5 stimulates the histone methyltransferase activities of KMT2A, KMT2B, KMT2C, KMT2D, SETD1A, and SETD1B [29, 30]. Studies have shown that RBBP5 is associated with many cancers [13–16], such as prostate cancer [13], hepatocellular carcinoma [14], and gliomas [16]. These results indicate that the expression of RBBP5 was upregulated and played a crucial role in the progression of tumors. Liu et al. reported that although RBBP5 was highly expressed in multiple myeloma cells and associated with cell proliferation, RBBP5 knockdown resulted in an increased adhesion rate of multiple myeloma cells [15]. Liu et al. also showed that multiple myeloma cells become less sensitive to chemotherapy drug-induced apoptosis when RBBP5 expression is knocked down [15]. These results indicate that RBBP5 may play an important anticancer role. However, the role of RBBP5 in melanoma cancer remains unclear. In our study (Figures 2(a)–2(h)), we verified that the expression of RBBP5 was downregulated in melanoma tumor tissues and melanoma cells; furthermore, in our IHC results, the IHC score of positive RBBP5 staining in melanoma tumors was lower than the IHC score in adjacent tissues and nevi tissues. These results were inconsistent with previous reports. To explore the function and mechanism of RBBP5 in melanoma, we constructed stable melanoma cell lines A375 and A2058 by knocking out and overexpressing RBBP5 (Figures 2(i)–2(n)). We revealed that knockdown of RBBP5 in melanoma cells promoted proliferation, clone formation, invasion, and migration (Figure 4). However, overexpression of RBBP5 inhibited all of the above abilities (Figures 5(a)–5(j)). We also found that RBBP5 overexpression inhibited the cell cycle transition from the G0/G1 phase to the S phase (Figures 5(k)–5(m)). Thus, our study clearly proved that RBBP5 is a tumor progression inhibitor in melanoma.

WSB2 (WD repeat and SOCS box containing protein 2), as an E3 ubiquitination enzyme, has been demonstrated to play a key role in the proliferation of melanoma [26]. We used the STRING website (https://cn.string-db.org/) to predict proteins that could interact with WSB2. We found and demonstrated that WSB2 can bind with RBBP5 and downregulate the expression of RBBP5 (Figure 1). Thus, we considered that RBBP5, as a downstream factor, may play a key role in WSB2-mediated ubiquitination degradation and may be used as a substrate. Therefore, further research and verification are needed in the follow-up.

H3K4me is a well-known histone modification mediated by the SET 1 complex, which is stimulated by the WRAD (the components are RBBP5, WDR5, ASH2L, and DPY30) complex [31–33]. Furthermore, Li et al. reported that human RBBP5-ASH2L heterodimer is the major structural unit that interacts with and activates MLL family [33]. High levels of mono-, di-, and trimethylation of H3K4 (H3K4me1, H3K4me2, and H3K4me3) are detected as the promoters of genes and are associated with the transcriptional activity of genes [34–36]. Chen et al. first reported that broad H3K4me3 that was specifically enriched at the transcription start site could elongate the transcription of tumor suppressor genes and increase their activity [24]. In 2017, the results of the two studies were consistent with the results of previous studies; simultaneously, the mechanism was also described [37, 38]. Two studies of renal carcinoma showed that lower levels of H3K4me3 were correlated with advanced stage, distant metastasis, and a shorter period of progression-free survival [19, 20]. Han et al. reported that higher levels of H3K4me3 could improve the overall survival of ovarian cancer and inhibit the proliferation of ovarian cancer cells and could be a potential prognostic factor [22]. Our study conclusion agreed with previous studies showing that overexpression of RBBP5 can enhance the level of H3K4me3 and inhibit the progression of melanoma in vivo and in vitro. In contrast, other previous studies verified that the expression of H3K4me3 was high and associated with poor prognosis in cervical cancer [18], hepatocellular carcinoma [23], and colon cancer [21]. The discrepancy between these studies may be due to the broad H3K4me3 domain activating cell-type-specific or disease-specific genes [24, 39–41].

H3K4me3 was used to identify novel tumor suppressors because tumor suppressor genes (TP53, PTEN, GPX3, and SPRY2) were associated with broad H3K4me3 peaks [24]. Yang et al. showed that inhibiting the demethylation of H3K4me3 resulted in increased trimethylation of H3K4 and increased recruitment of H3K4me3 to the promoters of p16 and p27, thus increasing their transcription in breast cancer [17]. This conclusion is in accordance with two previous studies on lung cancer and gastric cancer [42, 43]. In our study, we demonstrated that H3K4me3 can directly bind to the promoter of p16 (Figure 3(g)). Therefore, knockdown of RBBP5 expression can decrease the trimethylation level of H3K4, thus resulting in decreased transcription of the tumor suppressor gene p16 (Figures 3(a)–3(c)). In contrast, overexpression of RBBP5 had the opposite effect (Figures 3(e)–3(g)). Our findings reveal a novel link between RBBP5, H3K4me3, and p16 and melanoma progression.

We further elucidated that the downstream signalling pathway correlated with RBBP5 tumor suppressor gene function in melanoma and verified that RBBP5-mediated tumor inhibition could be, at least partly, due to inhibition of Wnt/*β*-catenin signalling, which activation is important for T-cell exclusion, resistance to antiPD-L1/antiCTLA4 therapy, and progression of melanoma [26, 44, 45]. RBBP5 knockdown induced Wnt/*β*-catenin signalling activation in A375 and A2058 cells, as evidenced by western blotting, and the expression of Wnt/*β*-catenin signalling representative genes (*β*-catenin, c-myc) was upregulated. In our study, we also found that RBBP5 knockdown induced EMT, and the expression of N-cadherin and MMP-7 was upregulated, while the expression of E-cadherin was downregulated (Figure 6). Puisieux et al. [46] reported that EMT could be induced by Wnt/*β*-catenin to stimulate several EMT-related factors, such as Snail, ZB3, and E47. Because the occurrence and development of cancer is a complex process, a variety of signalling pathways are involved and interact. The mechanism of the interaction of Wnt/*β*-catenin and EMT should be further explored.

The epithelial-mesenchymal transition (EMT) describes a reversible switch from an epithelial-like to a mesenchymal-like phenotype [47–50]. EMT was crucial for the invasion and metastasis of tumor, which has been suggested as a driving role in the acquisition of a metastatic phenotype [51–54]. A determinant hallmark of EMT is the presence of the cadherin switch. The epithelial to neural cadherin switch has been reported by previous studies, which was accomplished by the downregulation of the protein E-cadherin and upregulation of N-cadherin [55–57]. In our study, we observed that partial-EMT-related markers N-cadherin and MMP-7 were upregulated, and E-cadherin was downregulated after RBBP5 knockdown in melanoma cells, which agree with previous research about cadherin switch.

EMT-related markers include N-cadherin, E-cadherin, vimentin, and Snail, which have been documented in lots of cancers, such as breast [58], colorectal [59], oral [60], lung [61], pancreatic [62], melanoma [51, 63–65], and liver cancers [66]. EMT-related markers were associated with tumor initiation, invasion, metastasis, and resistance to therapy [47, 67–69]. Several previous studies have reported that the membranous E-cadherin/*β*-catenin complexes were associated with tumor progression and poor survival [70, 71]. Luo et al. reported that the high expression of nuclear vimentin and cytoplasmic E-cadherin was significantly associated with worse outcome of nasopharyngeal carcinoma [47]. Deeb et al. also demonstrated that cytoplasmic high staining of E-cadherin was associated with shorter survival in lung cancers [72]. Vimentin is another mesenchymal marker for EMT, and several reports have demonstrated that vimentin is overexpressed in cancers, such as gastric cancer [73], breast cancer [74], and prostate cancer [75], which is associated with invasive phenotype and poor prognosis. The prognostic effect of N-cadherin was also contradictory in some studies. Bachmann reported that N-cadherin expression has no correlation with the clinical outcome in melanoma patients [63], but Kreizenbeck et al. showed that high expression of N-cadherin was associated with better overall survival [64]. On the contrary, Pieniazek found that high expression of N-cadherin was associated with shorter overall survival [65]. Lade-Keller et al. reported that when a “switch profile” (E-cadherin switches to N-cadherin expression) is triggered, it is significantly associated with poor survival and distant metastasis-free survival in melanoma patients [76]. Despite the different EMT states and mechanisms regulating cell in melanoma reported, there are still many questions that are unclear. Combining the previous research and our research, we can find that EMT-related markers are closely linked with the prognosis of many types of cancer, which indicate that further studying new therapeutic strategies related to the control mechanism of EMT can help us prevent melanoma progression and metastasis, providing a new promising therapeutic method in the future.

## 5. Conclusions

**Figure 9:**
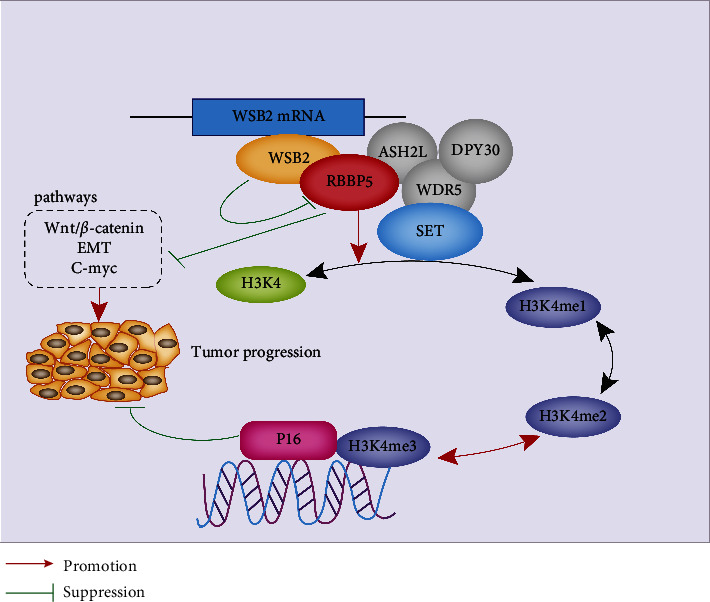
A model of the biological function and mechanism of RBBP5 in melanoma.

Altogether, our study demonstrated the potential ability of RBBP5 to act as a tumor suppressor to inhibit the progression of melanoma by inhibiting the Wnt/*β*-catenin signalling pathways and EMT. Furthermore, our research provided strong evidence that RBBP5 can increase the expression of H3K4me3, which could promote the transcription of p16 by directly binding to the promoter of p16 and inhibiting the progression of melanoma (Figure 9). Thus, our findings suggest that RBBP5 might be a potential therapeutic target to reverse melanoma progression.

## Data Availability

All data generated or analyzed during this study are included within the article. The datasets used during the present study are available from the corresponding author on reasonable request.

## Supplementary Materials

Supplementary Materials Supplementary Figure S1: expression of related proteins after knockdown of RBBP5 in mouse xenograft tumor tissues. Supplementary Figure S2: expression of related proteins after overexpression of RBBP5 in mouse xenograft tumor tissues. Figure S3: the body weight of the mice after knockdown of RBBP5 or overexpression.
