# Supplementary material for: Retinoblastoma-Binding Protein 5 Regulates H3K4 Methylation Modification to Inhibit the Proliferation of Melanoma Cells by Inactivating the Wnt/β-Catenin and Epithelial-Mesenchymal Transition Pathways
Source: J Oncol. 2023 Feb 21;2023:5093941. doi: 10.1155/2023/5093941 (PMC9974310; doi:10.1155/2023/5093941)
Supplement: Supplementary Materials — Supplementary Figure S1: expression of related proteins after knockdown of RBBP5 in mouse xenograft tumor tissues. Supplementary Figure S2: expression of related proteins after overexpression of RBBP5 in mouse xenograft tumor tissues. Figure S3: the body weight of the mice after knockdown of RBBP5 or overexpression. [file 5093941.f1.pdf]

Supplementary Figures

Supplementary Figure S1: Expression of related proteins after knockdown RBBP5 in Mouse xenograft tumour tissues.

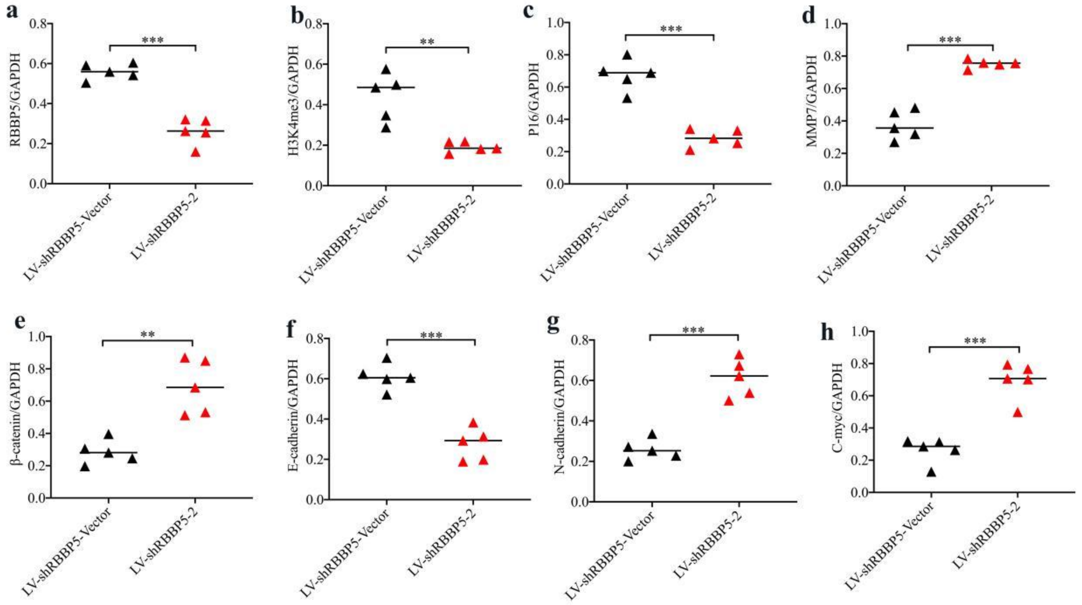

Supplementary Figure S1: Expression of related proteins after knockdown RBBP5 in Mouse xenograft tumour tissues. (a-c) Mouse xenograft tumour tissues were used to verify the expression of the downstream proteins H3K4me3 and p16 by Western blotting. (d-h) Western blotting was used to demonstrate the expression of β-catenin and c-myc in the Wnt/β-catenin signalling pathway and N-cadherin, E-cadherin, and MMP-7 in EMT. Data are represented as the mean ± SD. *, p<0.05, **, p<0.01, ***p<0.001; ns, no significance.

Supplementary Figure S2: Expression of related proteins after overexpression RBBP5 in Mouse xenograft tumour tissues.

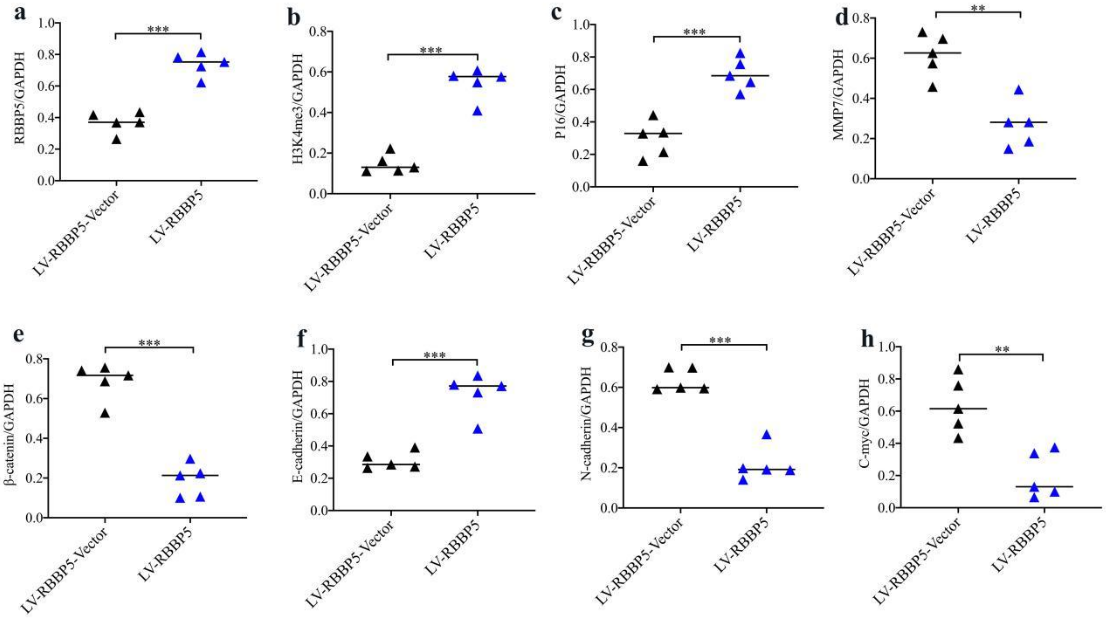

Supplementary Figure S2: Expression of related proteins after overexpression RBBP5 in Mouse xenograft tumour tissues. Tumor tissues from 5 mouse in each group were used for

Western blotting to confirm expression of related proteins after overexpression RBBP5 in A375 cells. (a-c) Mouse xenograft tumour tissues were used to verify the expression of the downstream proteins H3K4me3 and p16 by Western blotting. (d-h) Western blotting was used to demonstrate the expression of β-catenin and c-myc in the Wnt/β-catenin signalling pathway and N-cadherin, E-cadherin, and MMP-7 in EMT. Data are represented as the mean ± SD. *, $p<0.05$, **, $p<0.01$, ***$p<0.001$; ns, no significance.

Supplementary Figure S3: The body weight of the mice after knockdown RBBP5 or overexpression.

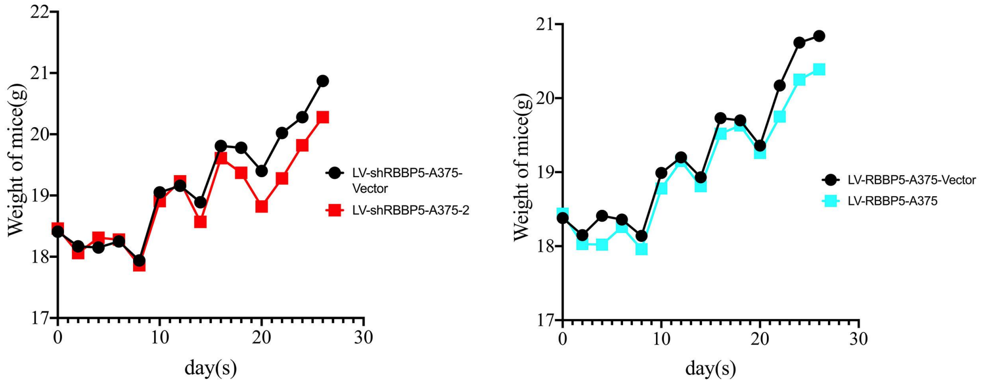

Figure S3: The body weight of the mice after knockdown RBBP5 or overexpression Each mouse (each Group n = 10 biologically independent samples) was subcutaneously injected with 2 × 10^6 A375 cells into the black (LV-shRBBP5-Vector), red (LV-shRBBP5-2) and blue (LV-shRBBP5). Weight changes were measured every two days, at the end of the experiment, The body weight of the mice are shown (S3). There was no significant statistical significance between two groups.
